# Tackling the Antibiotic Resistance Caused by Class A *β*-Lactamases through the Use of *β*-Lactamase Inhibitory Protein

**DOI:** 10.3390/ijms19082222

**Published:** 2018-07-30

**Authors:** Warawan Eiamphungporn, Nalini Schaduangrat, Aijaz Ahmad Malik, Chanin Nantasenamat

**Affiliations:** 1 Department of Clinical Microbiology and Applied Technology, Faculty of Medical Technology, Mahidol University, Bangkok 10700, Thailand; warawan.eia@mahidol.ac.th; 2 Center of Data Mining and Biomedical Informatics, Faculty of Medical Technology, Mahidol University, Bangkok 10700, Thailand; nalini.schaduangrat@gmail.com (N.S.); ajaz_me@hotmail.com (A.A.M.)

**Keywords:** *β*-lactamase, *β*-lactamase inhibitor protein, antibiotic resistance

## Abstract

β-Lactams are the most widely used and effective antibiotics for the treatment of infectious diseases. Unfortunately, bacteria have developed several mechanisms to combat these therapeutic agents. One of the major resistance mechanisms involves the production of β-lactamase that hydrolyzes the β-lactam ring thereby inactivating the drug. To overcome this threat, the small molecule β-lactamase inhibitors (e.g., clavulanic acid, sulbactam and tazobactam) have been used in combination with β-lactams for treatment. However, the bacterial resistance to this kind of combination therapy has evolved recently. Therefore, multiple attempts have been made to discover and develop novel broad-spectrum β-lactamase inhibitors that sufficiently work against β-lactamase producing bacteria. β-lactamase inhibitory proteins (BLIPs) (e.g., BLIP, BLIP-I and BLIP-II) are potential inhibitors that have been found from soil bacterium *Streptomyces* spp. BLIPs bind and inhibit a wide range of class A β-lactamases from a diverse set of Gram-positive and Gram-negative bacteria, including TEM-1, PC1, SME-1, SHV-1 and KPC-2. To the best of our knowledge, this article represents the first systematic review on β-lactamase inhibitors with a particular focus on BLIPs and their inherent properties that favorably position them as a source of biologically-inspired drugs to combat antimicrobial resistance. Furthermore, an extensive compilation of binding data from β-lactamase–BLIP interaction studies is presented herein. Such information help to provide key insights into the origin of interaction that may be useful for rationally guiding future drug design efforts.

## 1. Introduction

Since the discovery of the first commercially successful antibiotic, penicillin, by Alexander Fleming in 1928, the threat of antimicrobial resistance (AMR) has been looming. The resistance occurs naturally and thus, can only be slowed but not completely stopped. However, the misuse and abuse of antibiotics in veterinary and human medicine have accelerated the process. AMR causes longer hospital stays, higher medical costs and increased mortality making it a serious global health concern [1]. Recently, it was reported that more than 700,000 deaths worldwide can be attributed annually to AMR, which represents a low estimate due to inadequate reporting and surveillance [2]. Moreover, it has been estimated that AMR could cost 100 trillion USD between now and 2050 with the annual death toll reaching 10 million over that period [3]. Therefore, the World Health Organization has emphasized the urgency in the discovery of new antibiotics [4]. However, a major drawback of the drug development process is its time consuming nature and the fact that no new classes of antibiotic drugs have been identified since 1984. Although several peptide-based antimicrobials, i.e., teixobectin [5] have been discovered, they are not applicable for clinical use. In addition, the phenomenon of AMR has been further exacerbated due to the overuse/misuse of antibiotic drugs (i.e., easy availability over the counter, use in agriculture and food production, etc.) [6]. The effect of AMR on human health occurs when appropriate antimicrobial drugs (i) are not available or are not yet in existence or (ii) they are available but are of poor quality or come at a prohibitively high cost to individuals and society. Furthermore, the two main problems that compromise the efficacy of antibiotics include the rapid emergence and dissemination of antibiotic resistance among bacteria and the growing gap between increased AMR and the development of new and effective molecules to combat it [7].

**Figure 1:**
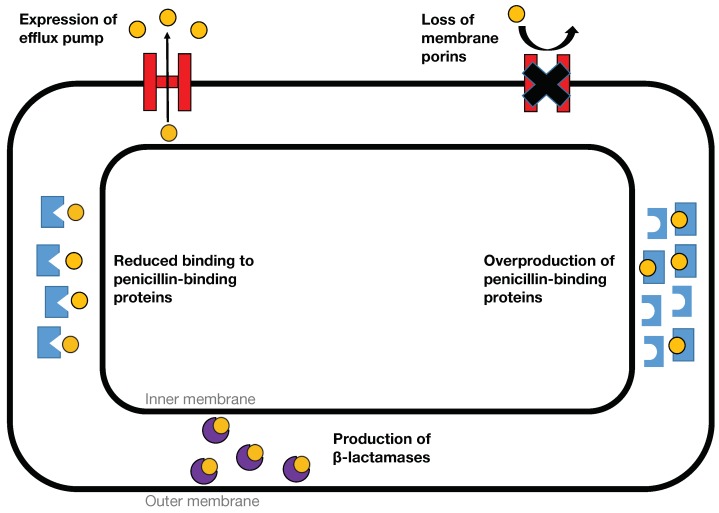
General mechanisms of β-lactam antimicrobial resistance. The peptidoglycan cell wall is not shown for simplicity. Compounds are shown as yellow circles while proteins are shown as either red or blue to denote their involvement in the antimicrobial resistance of Gram-negative and Gram-positive bacteria, respectively, while the purple color indicates their involvement in both Gram-negative and Gram-positive bacteria. The β-lactam resistance in Gram-negative bacteria can occur through three mechanisms: (i) production of β-lactamases (i.e., the most common mechanism) for β-lactam drug degradation, (ii) increase of the efflux pump expression to expel drugs and (iii) decrease in porin expression to reduce the drug’s uptake. Meanwhile, the β-lactam resistance in Gram-positive bacteria can arise via prevalent mechanisms, such as the alteration of penicillin-binding proteins (PBPs) to reduce the binding affinity between drugs and PBP targets, the overproduction of PBPs to replace the drug binding PBPs and production of β-lactamases (i.e., less frequent compared to Gram-negative bacteria) to destroy the drugs. Figure adapted from reference [17].

AMR can be found in both Gram-positive and Gram-negative bacteria. Bacterial resistance can arise in all antibiotic classes via several mechanisms (i.e., the degradation or modification of antibiotics via enzymes, modification of the antibiotic target site and prevention of access to the target site by permeability alteration or forceful efflux) [8]. β-Lactams (e.g., penicillins, cephalosporins, carbapenems and monobactams) are the most successful class of antibiotics, constituting 60% of worldwide antibiotic usage, and are among the most effective agents for treatment of infectious diseases [9]. They kill bacteria by inactivating the penicillin binding protein (PBP)-II, which represents a class of enzymes with essential roles in the synthesis of bacterial cell wall that ultimately leads to cellular death [10,11]. This class of antibiotics boasts many benefits such as ease of delivery, low toxicity, potent activity, and small cost that translate into its wide availability [12]. However, like most antibiotics, β-lactam resistance can occur through multiple molecular mechanisms including the production of efflux pumps, modification or reduced production of outer membrane porins, alterations of PBPs (i.e., the molecular target of β-lactams) and the production of β-lactamase for inactivating antibiotics [13]. The general mechanisms of β-lactam resistance, as achieved by both Gram-positive and Gram-negative bacteria, are shown in Figure 1.

**Figure 2:**
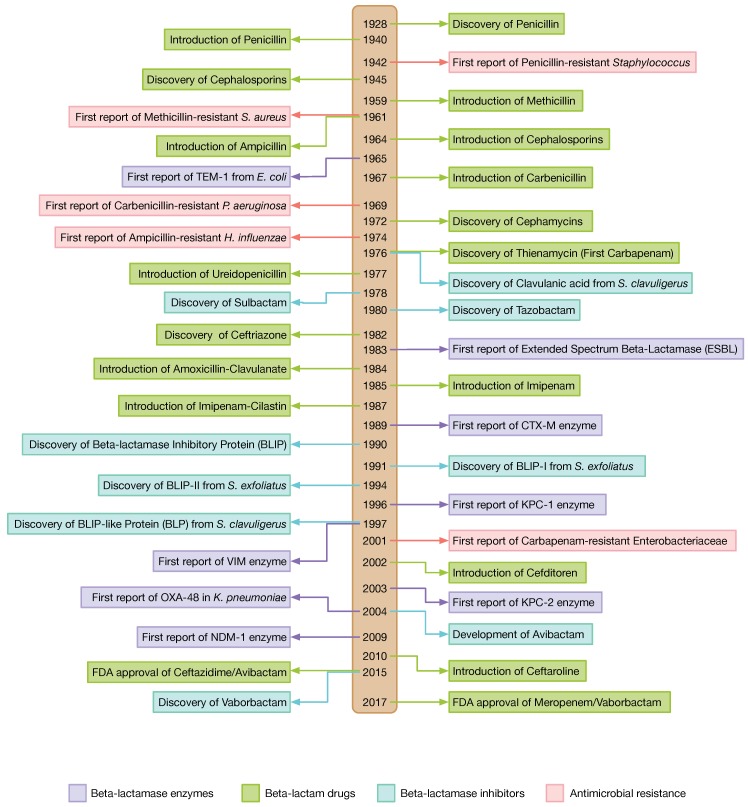
Timeline of the discovery of the β-lactam drug and the β-lactamase inhibitor against the development of their resistance.

Notably, the major mechanism of resistance against β-lactams in Gram-negative bacteria is the synthesis of β-lactamases, which irreversibly open the β-lactam ring of antibiotics [14]. The β-lactamase enzymes produced by bacteria are diverse and can be categorized into several classes. One of the strategies to overcome β-lactamase-mediated resistance is the development of β-lactamase inhibitors (BLIs). These small molecule inhibitors were discovered and have been applied in combination with β-lactams for efficient therapy. In addition, the popularity of peptide-based drugs has gained momentum in the last decade compared to small molecule drugs. This is due to the fact that, for peptide inhibitors, it is easier to produce a large collection of peptides with various properties for screening purposes using methods such as phage display or peptide array [15]. Moreover, peptide drugs are more selective; however, they exhibit poorer pharmacokinetic properties compared to small molecules. Furthermore, their short half-life can be advantageous in preventing drug resistance [16]. The timeline of the discovery of β-lactam drugs and β-lactamase inhibitors and the development of resistance towards them is shown in Figure 2.

Herein, this systematic review focuses on the current state-of-the-art of research on β-lactamase inhibitory proteins (BLIPs) with an emphasis on their binding mechanism with β-lactamases. BLIP constitutes an interesting class of protein-based inhibitor of β-lactamases which, in recent years, attracted great attention owing to its robust properties and effectiveness. To the best of our knowledge, this article represents the first review on the potential of BLIPs as a promising venue for tackling antibiotic resistance. It is hoped that this review will contribute to further growth of the field by providing the necessary first steps towards understanding this exciting field of research.

## 2. β-Lactamases

β-Lactamases (BLs) represent one of the most common causes of bacterial resistance to β-lactam antibiotics, particularly in Gram-negative bacteria [18]. These enzymes can inactivate almost all β-lactam antibiotics by binding covalently to their carbonyl moiety and hydrolyzing the β-lactam ring. The substrate spectrum of β-lactamases has increasingly been broadened. β-Lactamases are produced extracellularly by Gram-positive bacteria and in the periplasmic space by Gram-negative bacteria. Genes for β-lactamase enzymes can be found on the bacterial chromosome and are also often present on mobile genetic elements, such as plasmids and transposons. Bacterial β-lactamases are members of an enzyme family (EC 3.5.2.6) and are further divided based on two major classification schemes of β-lactamases (i.e., Ambler classification and Bush classification). The Ambler classification [19] is based on amino acid sequence similarities (protein homology), while the Bush classification [20] is based on functionality (substrate and inhibition profile). According to the Ambler classification, β-lactamases can be further sub-divided into four molecular classes (i.e., A–D) where classes A, C and D utilizes a serine moiety while class B being composed of a metalloenzymatic zinc ion at its active site. Based on the Bush classification, three major groups are formed: Group 1, cephalosporinases; Group 2, serine β-lactamases; and Group 3, metallo-β-lactamases (MBL). Several new subgroups of each of the major groups have been described based on specific attributes of individual enzymes. As of now, β-lactamases include >2000 unique, naturally occurring amino acid sequences [21]. In addition, among Gram-negative bacteria, the emergence of extended spectrum β-lactamases (ESBLs) has been a major concern [22]. ESBLs are characterized for their ability to hydrolyze third and fourth generation cephalosporins and monobactams but not cephamycins and carbapenems [23]. Most of the ESBLs are of molecular Class A, with the exception of OXA-type enzymes (Class D β-lactamases or oxacillinase) [24]. They are inhibited by β-lactamase inhibitors, such as clavulanic acid [25].

### 2.1. Class A β-Lactamase

Class A enzymes are the most commonly identified among clinical isolates and exist in both Gram-positive and Gram-negative bacteria. Class A β-lactamases show broad substrate hydrolysis profiles, including penicillins, cephalosporins and, for a few enzymes, carbapenems [26]. TEM-1 β-lactamase is a class A enzyme (i.e., named after the patient Temoneira from which the isolate was taken from) and is the most prevalent plasmid-encoded β-lactamase in Gram-negative bacteria [27]. TEM-1 can hydrolyze penicillins and early generation cephalosporins. Numerous other class A β-lactamases, such as SHV-1 (sulfhydryl variable), SME-1 (*Serratiamarcescens* enzyme), Bla1 (β-lactamase type 1) and PC1 (penicillinase type 1) have substrate profiles similar to those of TEM-1 and are found in various Gram-positive and Gram-negative bacteria [20]. Notably, TEM-1, SHV-1 and Bla1 are β-lactamases that share at least 30% identity [28], and mutations of the aforementioned enzymes are responsible for ESBL phenotypes. Additionally, there are other types of class A β-lactamases, such as CTX-M (cefotaximase) and KPC (*Klebsiellapneumoniae* carbapenemase), that should be of great concern since they are widely spread and cause ESBL phenotypes, with CTX-M being the most common [29,30]. Furthermore, CTX-M β-lactamases have high hydrolysis activity against cefotaxime and are capable of higher cephalothin hydrolysis in comparison to penicillins, cefaloxine and ceftazidime [31]. KPC can hydrolyze almost all β-lactams, including penicillins, cephalosporins, monobactams and carbapenems [32]. However, Class A enzymes can be inhibited by β-lactamase inhibitors, such as clavulanic acid, tazobactam and sulbactam. Among Class A β-lactamases, the KPC enzymes are of great concern as they confer resistance to all β-lactams and are not efficiently inhibited by typical Class A inhibitors [32]. In addition, KPC-2 and KPC-3 are the most widespread variants of KPC [33,34].

**Figure 3:**
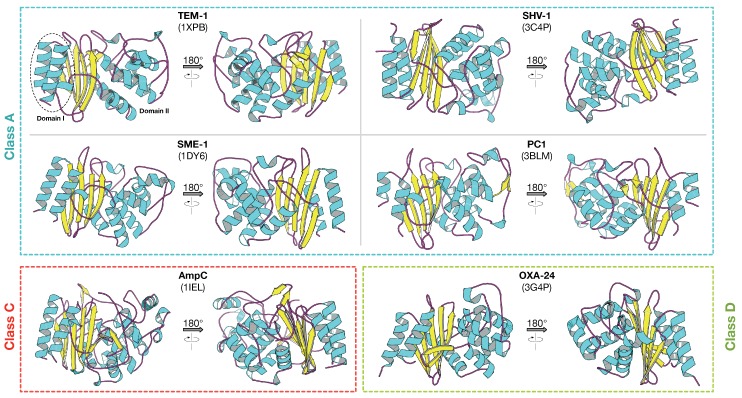
3-D structure of serine β-lactamases, i.e., A, C and D. Alpha-helices are represented as a cyan ribbon (inner face shown in grey), beta-strands are shown in yellow and loops are in magenta. Each structure is labeled by its common name followed by the PDB (Protein Data Bank) ID in parenthesis on the subsequent line. Each structure is rotated by 180${}^{\circ }$ for better understanding of structural details.

One of the characteristic feature of Class A β-lactamases is that they are comprised of four important structural motifs, Ser70-X-X-Lys73, Ser130-X-Asn132, Lys234-Thr/Ser-Gly, and the Ω-loop which are found around the active-site pocket and are conserved in all members of Class A β-lactamases. The general enzymatic mechanism of action of this class of enzymes involves nucleophilic attack by Ser70 on carbonyl carbon of the β-lactam ring, followed by the formation of an acyl-enzyme intermediate. Site-directed mutagenesis has proved that replacing the active site Ser70 with other amino acids results in the inactivation of enzymatic activity [35]. Furthermore, it has been proposed that Lys73 and Glu166 act as the general bases in the acylation or the deacylation step, while Lys73, Lys234 and Ser130 are involved in the formation of a hydrogen bond network with a water molecule that is important in the deacylation step [36]. The crystal structures of Class A β-lactamases, such as TEM-1 (PDB ID: 1XPD), SHV-1 (PDB ID: 3C4P), SME-1 (PDB ID: 1DY6), Bla1 (PDB ID: 3QHY) and PC1 (PDB ID: 3BLM), have already been elucidated. The overall structure of Class A β-lactamases is mostly conserved and consists of two main domains. Domain I is mainly composed of α-helices while Domain II is comprised of five β-strands containing α-helices, as shown in Figure 3.

### 2.2. Class B β-Lactamase

**Figure 4:**
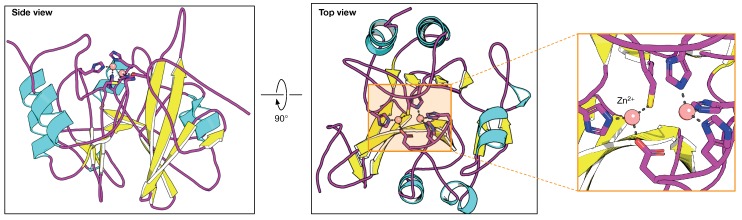
3-D structure of the metallo-β-lactamase NDM-1 (PDB ID: 4HL2) in cartoon representation showing interaction with Zn${}^{2+}$ ions (depicted as an orange sphere). A zoomed-in view of the Zn${}^{2+}$ ions interacting with surrounding residues is also shown.

The Class B enzymes or metallo-β-lactamases have a broad substrate spectrum and can catalyze the hydrolysis of all β-lactams with the exception of monobactams. Moreover, they are not inhibited by β-lactamase inhibitors, such as clavulanate, sulbactam or tazobactam that are effective against serine-based β-lactamases [37]. However, Class B enzymes are inactivated by metal chelators, such as ethylenediaminetetraacetic acid (EDTA) [38]. There are several types of Class B β-lactamases, including VIM (Verona integron-encoded metallo-β-lactamase), IMP (imipenemase) and NDM-1 (New Delhi metallo-β-lactamase). In the past, these enzymes were initially found to be chromosomally encoded and in non-pathogenic organisms. Nevertheless, the spread of the IMP- and VIM-type enzymes in Gram-negative pathogens including Enterobacteriaceae, *P. aeruginosa* and *A. baumannii* was reported in the 1990s [39]. In addition, the enzymes are encoded as gene cassettes within integrons and can insert into bacterial chromosomes or plasmids. This phenomenon leads to the their spread among bacterial species which can, in turn, lead to the emergence of multidrug resistant bacteria. NDM-1 is a novel metallo-β-lactamase that confers resistance to all β-lactams with the exception of aztreonam [40] and has been discovered recently in *K. pneumoniae* and *E. coli* [41]. Presently, NDM-1 resistant variants have spread globally, and bacterial isolates have frequently been detected worldwide. [42]. Furthermore, NDM-1 encoded genes are found on plasmids which can be transferred among Gram-negative bacteria thus making NDM-1 one of the most clinically significant β-lactamases [34]. Class B β-lactamases require zinc or other heavy metal ions for catalysis, as shown in Figure 4. These enzymes can be classified into three main subclasses: B1, B2 and B3. Both B1 and B3 have two zinc ions with a stable coordination bond at the zinc-binding site, while B2 has only one zinc ion that is tightly bound and the presence of another zinc ion reduces its enzymatic activity [43]. Even though their sequence identities are diverse, the overall crystal structures are very similar, with the possession of a characteristic αβ/βα sandwich fold comprising two central β-sheets and five α-helices on the external faces.The zinc-binding motifs in these scaffolds include six residues at the active site located at the external edge of the ββ sandwich. The zinc-binding motifs coordinate with either one or two zinc ions that are central to the catalytic mechanism [44].

### 2.3. Class C β-Lactamase

The Class C enzymes or AmpC type β-lactamases are commonly isolated from extended-spectrum cephalosporin resistant Gram-negative bacteria [37]. These enzymes are typically encoded on the chromosome and plasmids. Chromosome-mediated AmpC β-lactamases have been found in many Gram-negative bacilli, such as *P. aeruginosa*, *Enterobacter* spp., *Acinetobacter* spp., *Aeromonas* spp., *C. freundii*, *E. coli* and *S. marcescens* [45]. In most genera of Enterobacteriaceae, AmpC is inducible [46], unlike plasmid-encoded AmpC where the enzymes are almost always expressed constitutively [47]. The most commonly encountered plasmid-mediated AmpC β-lactamases belong to the CMY (cephamycinase), FOX (cefoxitinase), and DHA (Dhahran Hospital in Saudi Arabia) families [48]. The sequence of the *ampC* gene from *E. coli* was reported in 1981 [49] and was observed to differ from the sequence of penicillinase-type lactamases such as TEM-1, but, like TEM-1, it has serine at its active site [50]. In contrast to ESBLs, AmpC β-lactamases hydrolyze diverse β-lactam antibiotics, including cephamycins (e.g., cefoxitin and cefotetan), oxyimino cephalosporins (e.g., ceftazidime, cefotaxime and ceftriaxone) and monobactams (e.g., aztreonam). However, the hydrolysis rates for cefepime, cefpirome and carbapenems are very low. Furthermore, AmpC β-lactamases are not inhibited by EDTA or by Class A β-lactamase inhibitors, such as clavulanic acid, sulbactam and tazobactam. Moreover, AmpC β-lactamases are poorly inhibited by *p*-chloromercuribenzoate [46]. However, the enzymes are specifically inactivated by boronic acid and avibactam [48,51]. The 3-D structures of most Class C β-lactamases are relatively conserved and consist of two main domains, of which, Domain I has one helix and Domain II is composed of an αβ domain, as shown in Figure 3. Similar to Class A β-lactamases, Class C also have four conserved structural motifs around the active site pocket, such as Ser64-X-X-Lys67, Tyr150-X-Asn152, Lys315-Thr316-Gly317 and the Ω-loop [46]. The active site can be divided into two subsites: R1 representing the position of the β-lactam nucleus side chain C7 (or C6) in β-lactam antibiotics and R2 representing the opposite region interacting with the right part of the β-lactam ring, including the R2 side chain at C3 (or C2). The R1 subsite is surrounded by the Ω-loop, and the R2 subsite is enclosed by the R2-loop containing the α-10 and α-11 helices. The general mechanism of action is similar to other classes of β-lactamases, i.e., acylation of the serine residue by attacking the carbonyl carbon of the β-lactam ring to form an acyl-enzyme intermediate and deacylation of the acyl-enzyme thus releasing the hydrolyzed antibiotics [52].

### 2.4. Class D β-Lactamase

The Class D enzymes or OXA-type β-lactamases (oxacillinases) are widely disseminated in Gram-negative bacteria [53]. The OXA encoded genes are found on both chromosomes as well as in plasmids of diverse bacterial species, such as, *Acinetobacter* spp., *Pseudomonas* spp., *Burkholderia* spp., *Shewanella* spp. and Enterobacteriaceae [54]. Among the four β-lactamases molecular classes, Class D β-lactamases are the most diverse. Some OXA enzymes are restricted in their substrate profile (i.e., narrow spectrum), such as penicillins and the first generation of cephalosporins, while, in contrast, many OXA enzymes have extended their spectrum to late-generation cephalosporins and even carbapenems (broad spectrum) [55]. In addition, single amino acid substitutions are responsible for the expansion of their spectrum of activity in several groups of enzymes. Furthermore, since these enzymes are commonly linked to integrons and transposons, they can be transferred among species [53,56]. Nevertheless, Class D β-lactamases are classically described as being poorly inhibited by β-lactamase inhibitors, such as clavulanic acid, tazobactam and sulbactam [26]. However, this description is not precisely correct. Notably, both clavulanic acid and tazobactam exhibit inhibitory activity for some OXA enzymes [38].

On the whole, the crystal structures of most Class D β-lactamases (e.g., OXA-1, OXA-10, and OXA-13) are similar and consist of two main domains, of which Domain I is comprised of helices and the other domain is a mixed αβ domain including a central six-stranded antiparallel β-sheet, as shown in Figure 3. Besides having the classical conserved four structural motifs (i.e., Ser70-X-X-Lys73, Ser118-X-Val/Ile120, Lys216-Thr/Ser217-Gly218 and the Ω-loop) they also have two unique conserved motifs (i.e., Thr/Phe144-Gly145-Asn146 and Trp232-X-X-Gly235). Even though their overall enzymatic reaction is similar to that of other β-lactamase classes, their acylation and deacylation are facilitated by Lys73 which is unique to Class D β-lactamases [57].

## 3. β-Lactamase Inhibitors

**Figure 5:**
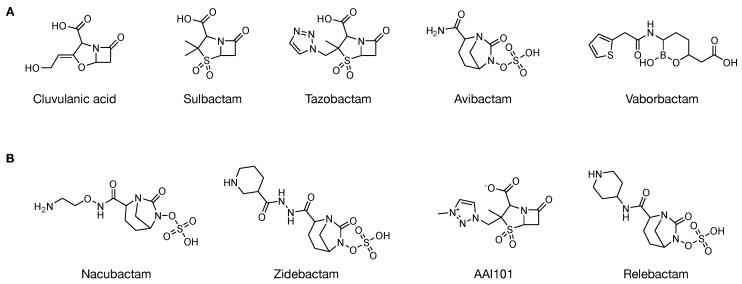
Chemical structures of β-lactamase inhibitors. Shown are those that have been FDA-approved (**A**) and those currently undergoing clinical trials (**B**).

As previously stated, the emergence of antibiotic resistance is a serious global public health concern. Such resistance against β-lactams is mediated by the production of β-lactamase enzymes, particularly the ESBL-producing bacteria. One strategy to combat the resistance is the use of combination therapies with β-lactamase inhibitors (BLIs). BLIs alone provide little intrinsic antibacterial activity and are usually prescribed with their β-lactam antibiotic counterpart. For example, the first BLI, clavulanic acid, is more potent when combined with amoxicillin. The inhibitors alter the substrates in their ability to assume long-lived, stable intermediates with β-lactamases and thus, help their partner β-lactam in inhibiting their PBP target. BLIs can bind and inactivate β-lactamases via reversible or irreversible mechanisms [38]. The chemical structures of Food and Drug Administration (FDA)-approved BLIs and those currently in clinical trials are shown in Figure 5.

### 3.1. Serine β-Lactamase (Class A, C and D) Inhibitors

#### 3.1.1. β-Lactam/β-Lactamase Inhibitors

The first β-lactamase inhibitor to be discovered was clavulanate (clavulanic acid) which was isolated from *S. clavuligerus* in 1977, followed by sulbactam and tazobactam in the 1980s [58,59,60]. Clavulanic acid is a clavam, whereas sulbactam and tazobactam are penicillanic acid sulfones [51]. They are small molecules and are structurally similar to penicillin but possess weak antibacterial activity on their own [9]. They irreversibly bind β-lactamases and protect the active antibiotics from inactivation when administered in combination with β-lactams. The first β-lactam/β-lactamase inhibitor combination that was approved by the FDA was augmentin (amoxycillin/clavulanate) [61]. In addition, the mechanisms of action for clavulanic acid, sulbactam and tazobactam are mainly restricted to type A Ser-β-lactamase, while having less effect on Class C enzymes and are essentially inactive against Class B and most Class D enzymes [62,63,64,65]. However, after three decades of clinical use, many resistant pathogen strains have been isolated.

#### 3.1.2. Non-β-Lactam/β-Lactamase Inhibitors

The first synthetic non-β-lactam/class A β-lactamase inhibitor was avibactam [66]. Its structure consists of a diaza-bicyclo octane (DABCO) core instead of the β-lactam core seen in tazobactam and sulbactam [67,68]. Ceftazidime–avibactam was approved by the FDA in 2015 [69]. Avibactam inhibits many serine-β-lactamases, including Class A, C and some Class D, by covalently binding and inhibiting the enzymes [70]. Notably, the inhibition mechanism of avibactam is unusual whereby the covalent inhibition proceeds in a similar fashion via the opening of the avibactam ring and the reaction is reversible, but deacylation results in regeneration of the intact compound as opposed to hydrolysis and turnover [67]. Following avibactam, other structurally-related β-lactamase inhibitors, including relebactam (MK-7655), nacubactam (FPI-1459, RG6080, OP0595) and zidebactam, have been developed and are currently in various phases of clinical trials [71]. Another class of non-β-lactam/β-lactamase inhibitor is the boronic acids which have been known as β-lactamase inhibitors for several decades. They act as competitive inhibitors, forming a tetrahedral intermediate by binding to the catalytic serine through a reversible, dative covalent bond [72]. The bound inhibitor mimics the tetrahedral structure of the high energy intermediate formed during β-lactam hydrolysis [73,74]. Several studies have demonstrated that different boronic acids are high affinity inhibitors of Class A, C and D β-lactamases [75,76]. Notably, there are limited OXA inhibitors because of the variability in the amino acid sequences of OXAs. However, boronic acid-based compounds and penicillin sulfone derivatives are promising candidates for the development of OXA-carbapenemase inhibitors [77,78]. A boronic acid-based inhibitor, vaborbactam, was recently approved by the FDA in 2017 in combination with meropenem [71]. Additionally, other novel non β-lactam inhibitors, such as ZINC01807204 and ZINC02318494, have been reported [79].

### 3.2. Class B β-Lactamase Inhibitors

The difficulty in finding a so-called ‘universal’ inhibitor of MBLs arises from the structural and mechanistic differences among the three subclasses of MBLs recognized so far and those that have been classified on the basis of structural similarity. All MBLs possess two potential zinc-binding sites and share a small number of conserved motifs bearing some of the residues that coordinate the zinc ion(s) [71]. MBL inhibitors are small molecules that can be classified into two types, zinc-dependent and zinc-independent inhibitors, based on their mechanisms [80]. The zinc-dependent inhibitors include zinc ion chelators, such as EDTA, and zinc binding compounds, such as thiols, dicarboxylates, hydroxamates, aryl sulfonamides, N-arylsulfonyl hydrazones and tetrazole-based compounds [81]. Recently, Zn-independent MBL inhibitors were reported, which act via a mode that does not involve direct zinc chelation but which may mimic interactions made by intact β-lactam substrates [82]. Unfortunately, currently, there are no clinically available metallo-β-lactamase inhibitors [83].

### 3.3. Resistance to β-Lactamase Inhibitors

Presently, the use of three classical β-lactamase inhibitors (e.g., clavulanic acid, tazobactam and sulbactam) in combination with β-lactam antibiotics is the mainstay of antibiotic therapy against Gram-negative bacterial infections. However, bacteria have evolved the mechanisms of resistance to overcome the inhibitory effects of these inactivators since these inhibitors share a common β-lactam core structure. The resistance occurs due to mutations of β-lactamase, particularly the amino acids at the active site, leading to ineffective binding and thus, reduced inhibition. Moreover, small molecule inhibitors in clinical use are rapidly degraded [84]. Of note, many β-lactamases are not inhibited by currently available inhibitors. Therefore, the search for novel β-lactamase inhibitors is needed to cope with these problems.

## 4. β-Lactamase Inhibitor Proteins

**Figure 6:**
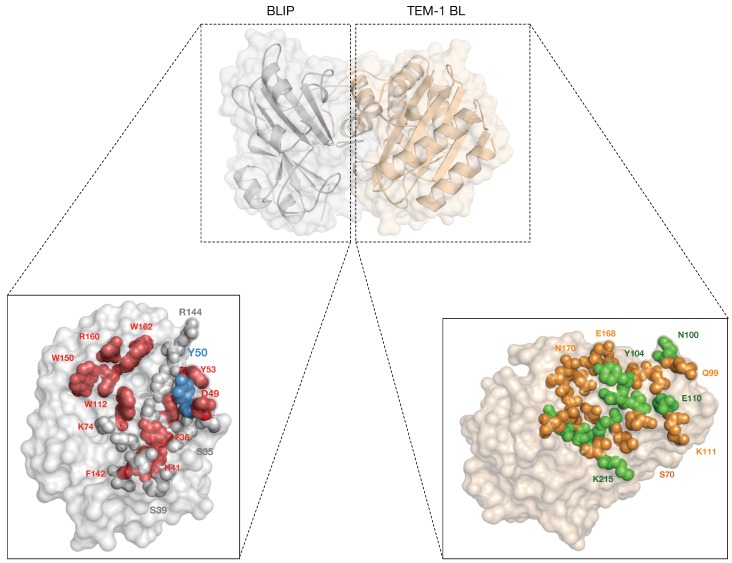
Illustration of A β-lactamase inhibitory protein (BLIP) (grey surface) bound to TEM-1 β-lactamase (wheat surface) from the X-ray structure (PDB ID 2B5R). (**A**) The interface is depicted by a spherical representation, while the non-interface is shown as a surface. The positions of the important residues on the BLIP structure that are substituted with varying effects on the binding affinity are shown as spheres and are colored in a gradient: red (significantly decreased the binding affinity), blue (increased binding affinity) and grey (no effect on binding affinity); (**B**) the contact residues on TEM-1-BLIP complex are colored in orange, while residues with hydrogen bonds are colored in green.

The β-lactamase inhibitor protein (BLIP) was first isolated from *S. clavuligerus* in 1990 [85]. BLIP represents a 17.5-kDa protein containing 165 amino acids with 36 amino acids as the signal peptide. It is secreted extracellularly and was characterized to inhibit β-lactamases from several bacterial species. In 1994, Strynadka et al. [86] determined the molecular structure of BLIP using the multiple isomorphous replacement (MIR) method which revealed BLIP to be a flat molecule, composed of a 76 amino acid domain in tandem repeat. Each domain contains a helix-loop-helix motif that packs against a four stranded antiparallel β-sheet. Additionally, kinetic analyses of BLIP with a wide spectrum of β-lactamases demonstrated that it is a potent inhibitor with the ability to inactivate Class A β-lactamase with inhibition constant (*K*${}_{i}$) values ranging from the micromolar to picomolar depending on the target enzyme. Particularly, BLIP binds and inhibits the TEM-1 β-lactamase (*K*${}_{i}$ = 0.5 nM) which is widely present in Gram-negative bacteria, and the KPC-2 β-lactamase (*K*${}_{i}$ = 1.2 nM) which hydrolyzes virtually all clinically useful β-lactam antibiotics [87,88]. Moreover, BLIP also weakly inhibits the penicillin binding protein (PBP) from *Enterococcus faecalis*. Furthermore, molecular docking and X-ray crystallography were employed to elucidate the protein–protein interaction (PPI) of BLIP and TEM-1 β-lactamase [89]. According to the crystallography study, the interface of interaction between BLIP and TEM-1 β-lactamase is one of the largest PPI which has also been extensively studied, as illustrated in Figure 6 [89]. The binding complex of this interaction consists of two hot spot residues (Asp49 and Phe142). In addition, the interface of the TEM–BLIP complex exhibited that there are 49 residues which make direct contact in the complex. These interactions include the two tandem repeat domains of BLIP which form a polar, concave surface that docks onto a predominantly polar, convex protrusion of the enzyme and two hairpin loops, thus allowing one loop from domain 1 of BLIP to insert into the active site of the β-lactamase. Notably, Asp49 is a key residue on one of these loops that forms four hydrogen bonds to the four conserved residues which are important for catalysis and substrate binding. In addition, Phe142 is involved in the stabilization of the inhibitory complex. The significance of these residues have been confirmed by site-directed mutagenesis [90], in which amino acids of interest are mutated to alanine followed by measurement of the *K*${}_{i}$ values. The results demonstrated that each mutation of BLIP increases the *K*${}_{i}$ for TEM-1 β-lactamase inhibition by approximately 100-fold. Moreover, the *K*${}_{i}$ of binding between wild-type BLIP and its two mutants to two TEM variants (Glu104Lys and Gly238Ser mutants) was also determined. The amino acids at positions 104 and 238 are located in the BLIP/β-lactamase interface. It was shown that the wild-type and both mutants of BLIP inhibit the Gly238Ser β-lactamase mutant at a similar level to that which they inhibit wild-type TEM-1 β-lactamase, whereas wild-type BLIP has a higher *K*${}_{i}$ for the Glu104Lys β-lactamase mutant, indicating that interactions between BLIP and β-lactamase residue Glu104 are important for wild-type levels of BLIP inhibition. Apart from this, two patches comprised of mainly aromatic residues are also involved in the binding of the complex (Patch 1: Phe36, His41 and Tyr53; Patch 2: Trp112, His148, Trp150, Arg160 and Trp162) [88].

Furthermore, electrostatic interactions are known to play a significant role in the binding specificity of the TEM–BLIP complex. The Glu73-Lys74 motif comes in contact with the Glu104-Tyr105 of TEM-1 by forming a salt bridge (i.e., Lys74 of BLIP with Glu104 of TEM-1), thus stabilizing the interaction. The importance of this salt bridge was determined with the replacement of Lys74 with Ala74 (i.e., via alanine scanning method) resulting in a 92-fold decrease in binding affinity. However, the same effect was not observed for alanine substitution of Glu73 [88]. In addition, Selzer et al. [91] proposed that electrostatic optimizations could result in an increased association rate (*K*${}_{on}$) between proteins. To that extent, they developed a program known as Protein Association Rate Enhancement (PARE) which determined the *K*${}_{on}$ of mutant complexes and the experimentally calculated wild-type via an energy based algorithm [91]. Moreover, by making use of the alanine-scanning mutagenesis method, Bogan and Thorn [92] determined that replacing the Ser and Thr residues with Ala on BLIP exhibited little to no effect on the binding affinity of PPI. However, these polar residues are still abundantly found on the binding interface, thus suggesting a role that is still unclear. This point has been reiterated in a recent publication in which Adamski and Palzkill [93] examined the effects of different amino acid (i.e., non-polar, small and polar residues) substitutions on the binding affinity of Tyr50.

Besides BLIP, two other protein based inhibitors, namely, BLIP-I and BLIP-II, have been discovered from *Streptomyces exfoliatus*. BLIP-I shares a 38% sequence identity with BLIP and inhibits β-lactamases with a similar mechanism based on structural and site-directed mutagenesis experiments. Of note, Asp49 of BLIP-I is identical to the 49th amino acid residue of BLIP that was characterized to be an essential residue for its inhibitory activity [94]. However, BLIP-I binds and inhibits the TEM-1 β-lactamase with a lower *K*${}_{i}$ compared to BLIP. BLIP-II on the other hand, is a 28-kDa protein that shares no sequence identity and has an unrelated structure to both BLIP and BLIP-I. A study of the BLIP-II crystal structure identified a seven-bladed propeller with a unique blade motif consisting of only three antiparallel strands. Interestingly, BLIP and BLIP-II demonstrate a similarity in their binding to TEM-1, even though BLIP-II does not share the same folding as BLIP [95]. Moreover, BLIP-II binds and inhibits the TEM-1 β-lactamase with a *K*${}_{i}$ value in the picomolar range. In addition, another BLIP homologue, named β-lactamase-inhibitory-protein-like protein (BLP), was discovered from *S. clavuligerus* [96]. This protein is predicted to be 154 amino acids in length, processed from a 182 amino acid precursor polypeptide and secreted extracellularly. Intriguingly, in spite of its apparent homology to BLIP (32% amino acid identity) and BLIP-I (42% amino acid identity), BLP does not possess detectable β-lactamase inhibitory activity and also lacks interaction with TEM-1. The apo BLP surface (non-TEM-1 binding) exhibits numerous favorable backbone–backbone/backbone–side-chain interactions with a protein partner that can be negated by the presence of a few, strongly unfavorable interactions, especially electrostatic repulsions [97].

A summary of the studies reporting the BL–BLIP interactions is provided in Table 1. As such, Strynadka et al. [86] were the first to identify BLIP interactions with the determination of its molecular structure. They were able to discern the uniqueness of BLIP, whereby it targets a single enzyme despite having two domains. In that same year, Kim and Lee [98] studied the interaction of BLIP-I and BLIP-II against Bacto Penase and concluded that BLIP-I shows a greater inhibition of the β-lactamase enzyme than BLIP-II. The next few years saw a rise in interaction studies conducted between BLIP and TEM-1 using a phage display system [15,99] and *E. coli* expression system [90,91,100,101,102,103]. For example, Albeck and Schreiber [100] studied the interaction of BLIP expressed in *E. coli* with its binding to TEM-1 in both the homogenous and heterogenous protein phases. The results showed that while BLIP is able to inhibit the catalytic activity of TEM-1, mutations on the TEM-1 active site have effects on the binding energy. In a similar fashion, Huang et al. [99] used phage display to alter the specificity of BLIP binding to TEM-1. The authors noted that with the use of this method, the bacteriophage containing the BLIP is able to bind specifically and with high affinity to β-lactamase. The authors took it a step further and also used the phage display method to identify the critical residues within the turn regions of BLIP that are specific for binding TEM-1 β-lactamase [101]. Besides TEM-1, BLIP also inhibits other class A β-lactamases, such as SHV-1, SME-1 and Bla1. Although SHV-1 shares a 68% sequence identity to TEM-1, its binding affinity to BLIP is approximately 2,200-fold lower than that of the TEM–BLIP complex. On the other hand, the sequence identity of SME-1 and Bla1 to TEM-1 is approximately 30%, but the inhibition of these subtypes is as strong as the inhibition of TEM-1 by BLIP [88,104].

As previously mentioned, the sequence homology of BLIP and BLIP-II does not exist; however, BLIP-II is still able to inhibit β-lactamase. To determine the affinity of the BLIP-II inhibition in comparison to BLIP for TEM-1 β-lactamase, Brown and Palzkill [105] used a M13 phage vector system in combination with enzyme inhibition assays which revealed that the BLIP-II TEM-1 interaction encompasses the same protruding loop-helix region of TEM-1 as is seen with BLIP. Nonetheless, both BLIPs bind to many of the same residues on the TEM-1 surface, as revealed by crystal structures [95,106]. BLIP-II, however, binds TEM-1 with an intensity of 150-fold tighter than BLIP, despite the fact that it has fewer contact residues and a smaller binding interface. Furthermore, according to the crystal structures, twelve TEM-1 residues are involved in interactions with both BLIP and BLIP-II out of a total of fourteen TEM-1 residues that interact with BLIP-II and twenty four TEM-1 residues that interact with BLIP [95]. Using alanine-scanning mutagenesis, Fryszczyn et al. [107] were able to determine that the impact of TEM-1 β-lactamase binding affinity for BLIP is lower than that for BLIP-II. Additionally, Brown et al. [108] used *K*${}_{on}$ and *K*${}_{off}$ interaction rate constants to investigate the potency of BLIP-II as an inhibitor of the KPC-2 carbapenemase. The authors observed a very tight interaction between BLIP-II and KPC-2 with a binding constant (*K*${}_{D}$) of 76 fM which is regarded as one of the tightest PPIs.

Taking it a step further, Chow et al. [87] explored the specificity of BLIP binding to KPC-2 by engineering a BLIP with amino acid substitutions targeted to β-lactamase. The authors were able to determine a winning combination of the K74T:W112D BLIP variant, which was shown by inhibition assays to retain high affinity for KPC-2 with three-fold tighter binding as compared to the binding with TEM-1 (20,000-fold weaker) and wild-type BLIP [87]. This infers the potential use of this binding as a enzyme sensor for drug resistance. Furthermore, to study the plasticity of the high-affinity binding in the BLIP–TEM interface, Cohen-Khait and Schreiber [109] introduced random TEM-1 mutations which were located on the protein surface, with the exception of one mutation (Thr29Glu). The BLIP was conjugated to a fluorescent protein (YPET) for fluorescence activated cell sorting (FACS) to determine the important residues for BLIP–TEM binding. Using deep sequencing, the authors determined that 11 out of 17 mutations involve charged residues which are known to play a vital role in determination of *K*${}_{on}$. Interestingly, all mutations except Thr29Glu showed no significant reduction in *K*${}_{on}$, but instead, 40% of those mutations provided a five-fold increase in *K*${}_{on}$ compared to the wild-type. In addition, only three of the amino acid residues did not tolerate mutagenesis (e.g., Gly236, Gly238, and Arg243). From previous literature, it was determined that Gly236 and Gly238 are important in regard to substrate specificity and are critical for tight BLIP binding [102]. Similarly, Arg243 of TEM-1 was shown to form a salt bridge with Asp49 on BLIP with a 10-fold decrease in binding affinity observed with its mutation [97]. More recently, Cohen-Khait and Schreiber [110] further extended their research by using a random TEM-1 yeast library to specifically select variants with faster *K*${}_{on}$ by using pre-equilibrium selection consisting of short incubation times (5 s to 30 min) and relatively low ligand concentrations (50 ng to 1 μM). The selection method was repeated twice in order to reduce the signal noise which arises due to the low ligand concentration used. The authors noted that their TEM-1 selected clones bound BLIP faster than the wild-types and the experimental results were, in general, in agreement with the computational predictions. Thus, the ability to select protein variants that can undergo faster association may prove useful in the drug development setting.

As a pioneer of BLIP–TEM-1 interaction studies, Strynadka et al. [89] utilized molecular docking software to predict the general mode of association between the TEM-1–BLIP binding complex and to also test the correlation between the then recently elucidated crystal structure in comparison to computationally-derived results. The authors noted that all of the molecular docking techniques were able to predict the general configuration of the BLIP–TEM-1 binding structure. Furthermore, Joughin et al. [111] identified non-contacting residues (i.e., residues that do not come in contact with the binding interface) that have significant impacts on the binding affinity of the TEM-1–BLIP interface. These identifications were made via consideration of intramolecular electrostatic interactions using methods based on a continuum solvation model which also takes into account the Van der Waals and hydrophobic contributions to the binding energetics. In addition, mutations of the identified residues to lysine were introduced as an opportunity to improve the electrostatic complementarity of BLIP for TEM-1. The authors elucidated four high-activity BLIP mutations (i.e., Asp133, Asp135, Gln161 and Asp163), out of which three mutations (i.e., Asp133, Asp135 and Gln161) were at a distance from the TEM-1 binding site where negligible changes in the Van der Waals binding free energy result (i.e., >0.1 kcal/mol as compared to wild-type) were observed. Nevertheless, the predicted free binding energy of these mutations were high in comparison to the wild-type. The aforementioned mutations were previously experimentally and computationally evaluated by Selzer et al. [112] using PARE computer software [91] to calculate the electrostatic interactions of their PPI associations and compare them to the experimentally determined *K*${}_{on}$ for the mutations. Meneksedag et al. [113] also evaluated the mutations of distant residues not in the binding site for TEM-1 and SHV-1 when bound to BLIP using molecular dynamic (MD) simulations. One of these regions was observed to be the H10 helix (i.e., acts as a barrier for the allosteric binding site) with a conserved Trp229 residue that interacts with two conserved Pro residues. MD simulations of the Trp229Ala mutation resulted in decreased stability for both TEM-1 and SHV-1. Furthermore, the binding affinity of BLIP towards TEM-1 and SHV-1 was determined by Kanlikilicer et al. [114] using MD simulations and MM-PBSA free binding energy calculations. The results showed that the binding of TEM-1 BLIP is tighter in comparison to the SHV-1 BLIP binding, which is in accordance with experimentally determined results [90,104]. Additionally, Fataftah et al. [115] conducted MD simulations and a dynamic cross-correlation map (DCCM) to study the distances of the C${}_{\alpha }$ atom in BLIP. The DCCM results provide a big picture analysis of high correlation among distant residues and should be further investigated.

## 5. Conclusions

Antimicrobial resistance is rapidly increasing and has become a major global health challenge. Infections caused by antimicrobial resistant pathogens are associated with high morbidity and mortality [132]. This problem could result in serious health consequences if efforts are not made to address the resistance. In spite of this increase in antimicrobial resistance, the development of new antimicrobial agents is declining and, as such, no new classes of antibiotics have been discovered since 1984. Currently, β-lactams are the most used class of antibiotics for the treatment of bacterial infections. Nonetheless, bacteria have evolved several resistance mechanisms for evading these therapeutic agents. Of particular note, one of the major mechanisms of β-lactam resistance is the production of β-lactamases. To overcome this obstacle, β-lactamase inhibitors have been developed and applied for therapy. These small molecule inhibitors of β-lactamases, such as clavulanic acid, sulbactam and tazobactam, have been employed in combination with β-lactams for clinical treatment. However, bacterial resistance to this kind of combination therapy has also been reported. Importantly, such small molecule inhibitors are rapidly degraded by β-lactamase [84]. Moreover, these traditional inhibitors have less effect on some class A β-lactamases, such as KPC enzymes and other classes of β-lactamases as well. Therefore, the design of novel inhibitors is an active area of research. Recent trends, such as peptide-based drugs, have gained interest as promising alternative strategies that afford remarkable advantages over small molecule drugs (i.e., target selectivity, ease to produce a large collection of peptides with various properties for screening purposes, ease of handling or storage and low level of induced resistance) [15,133]. As previously mentioned, peptides possess strong therapeutic potential but some inherent limitations (e.g., pharmacodynamics and kinetic properties) have restricted their applicability. Moreover, most peptides are not orally available and have a short half-life *in vivo* due to proteolytic degradation [134]. Even with these disadvantages, research on peptide bioactivity has been extensively performed and attempts are being made to address their limitations.

In the last few decades, proteinaceous β-lactamase inhibitors, particularly β-lactamase inhibitory proteins, have been discovered and extensively studied. These proteins are potent inhibitors that bind various Class A β-lactamases with high affinity, resulting in the inactivation of these enzymes. Interestingly, these proteins effectively bind and inhibit the KPC-2 enzyme, which is a significant clinical threat [28,108,135]. However, the therapeutic application of BLIPs is still limited due to their inability to penetrate into bacterial cells. In this regard, the protein inhibitors need to cross the cell wall and reach their intracellular target, β-lactamase. The inherent hydrophilic characteristic of proteins prevents their cellular uptake due to the hydrophobic nature of the cell wall and membranes [136]. To overcome this limitation, an alternative approach for the delivery of protein inhibitors in crossing the cell membrane and effectively reaching their target is through the use of cell-penetrating peptides as well as short cationic and amphipathic peptides. Taken together, the development of BLIPs could provide a promising avenue for the treatment of infections arising from Class A β-lactamase (i.e., ESBL or KPC producing bacteria).

Recent advancements in science and technology have led to the burgeoning of various omics, thereby amassing big data in the life sciences. Looking ahead, the big data available on β-lactamase–BLIP interactions could potentially be analyzed by bioinformatic tools to facilitate data-driven drug discovery efforts. Bioinformatics and computational intelligence have been instrumental in helping to unravel the hidden patterns of biological data and, as such, have afforded widespread adoption in the life sciences [137]. For instance, quantitative structure–activity relationships and proteochemometric modeling could potentially be employed (i) to make sense of such binding data as to pinpoint key residues that are involved in the inhibition of β-lactamase and (ii) to rationally design chemical structures or dictate the specific amino acid sequence in BLIP to afford robust inhibition of β-lactamase. These aforementioned approaches represent an underexplored area of research that has great potential to drive the design of robust drugs.

## Figures and Tables

**Table 1:** Summary of binding data from β-lactamase (BL)–BLIP interaction studies.

Year	BLIP Type	Protein Source	Binding Affinity	Detection Method ${}^{*}$	BL Target	Ref.
1994	BLIP	Secreted protein from *S. claviligerus*	$K_{i}$ = 18 pM − 12 μM	Enzyme inhibition	Class A-D and PBPs	[86]
1994	BLIP-I, BLIP-II	Secreted protein from *S. exfoliatus* SMF19	BLIP-I: $K_{i}$ = 0.062 nM, BLIP-II: *K*${}_{i}$ = 0.274 μM	Enzyme inhibition	Bacto Penase	[98]
1996	BLIP	Secreted protein from *S. claviligerus*	TEM-1: $K_{i}$ = 0.6 nM	Enzyme inhibition	TEM-1	[106]
1998	BLIP	Phage display system	IC${}_{50}$ = 1 nM	Phage ELISA	TEM-1	[99]
1999	BLIP	*E. coli* expression system	Enzyme inhibition: $K_{D}$ = 0.4 nM, FQT: $K_{D}$ = 0.3 nM, SPR: $K_{D}$ = 15 nM	Enzyme inhibition, FQT, SPR	TEM-1	[100]
1999	BLIP	*E. coli* expression system	TEM-1: $K_{i}$ = 0.11 nM, SHV-1: *K*${}_{i}$ = 1 μM	Enzyme inhibition	TEM-1, SHV-1	[90]
1999	BLIP	*E. coli* expression system	$K_{i}$ = 0.22 nM	Enzyme inhibition	TEM-1	[102]
2000	BLIP	*E. coli* expression system	$K_{i}$ = 0.12 nM	Enzyme inhibition	TEM-1	[101]
2000	BLIP-I	Secreted protein from *S. exfoliatus* SMF19 and *E. coli* expression system	Secreted protein: $K_{i}$ = 0.047 nM, Recombinant BLIP-I: *K*${}_{i}$ = 0.062 nM	Enzyme inhibition	TEM-1	[94]
2000	BLIP	*E. coli* expression system	*K*${}_{D}$ = 1.25 nM	Enzyme inhibition by SFF, SPR	TEM-1	[91]
2001	BLIP-II	Secreted protein from *S. exfoliatus* SMF19	$K_{i}$ = 0.0272 nM	Enzyme inhibition	TEM-1	[95]
2001	BLIP	Phage display system	BLIP peptide (residue C30-D49): $K_{i}$ = 352 μM	Enzyme inhibition	TEM-1	[15]
2002	BLIP	Secreted protein from *S. claviligerus*	$K_{i}$ = 2.76 nM	Enzyme inhibition	TEM-1	[103]
2003	BLIP	*E. coli* expression system	TEM-1: $K_{i}$ = 0.5 nM, SME-1: $K_{i}$ = 2.4 nM	Enzyme inhibition	TEM-1 and SME-1	[88]
2004	BLIP	*E. coli* expression system	$K_{on}$ = 4.4 ×10${}^{5}$ M${}^{-1}$·s${}^{-1}$	Enzyme inhibition by SFF	TEM-1	[116]
2004	BLIP	*B. subtilis* expression system	$K_{i}$ = 0.31 nM	Enzyme inhibition	TEM-1	[117]
2004	BLIP	*E. coli* expression system	TEM-1: $K_{i}$ = 0.5 nM, SME-1: $K_{i}$ = 2.4 nM, SHV-1: $K_{i}$ = 1 μM, Bla1: $K_{i}$ = 2.5 nM	Enzyme inhibition	TEM-1, SME-1, SHV-1 and Bla1	[104]
2006	BLIP	*E. coli* expression system	TEM-1: $K_{D}$ = 0.32 nM, SHV-1: $K_{D}$ = 1.25 μM	Enzyme inhibition	TEM-1, SHV-1	[118]
2007	BLIP	*E. coli* expression system	$K_{on}$ = 3.3 ×10${}^{4}$ M${}^{-1}$·s${}^{-1}$	Enzyme inhibition by SFF	TEM-1	[119]
2007	BLIP	*E. coli* expression system	TEM-1: $K_{i}$ = 0.5 nM	Enzyme inhibition	TEM-1	[120]
2008	BLIP	*E. coli* expression system	TEM-1: $K_{D}$ = 1.3 nM, SHV-1: $K_{D}$ = 1.72 μM	Enzyme inhibition	TEM-1, SHV-1	[121]
2009	BLIP, BLIP-I, BLIP-like protein	*E. coli* expression system	BLIP: $K_{i}$ = 0.1–0.6 nM, BLIP-I: $K_{i}$ = 0.05 nM	Enzyme inhibition	TEM-1	[97]
2009	BLIP	*E. coli* expression system	KPC-2: $K_{i}$ = 84 pM, KPC-3: $K_{i}$ = 250 pM	Enzyme inhibition	KPC-2, KPC-3	[28]
2009	BLIP	*E. coli* expression system	$K_{on}$ = 2×10${}^{5}$ M${}^{-1}$·s${}^{-1}$	Enzyme inhibition by SFF	TEM-1	[122]
2009	BLIP	*E. coli* expression system	TEM-1: $K_{i}$ = 0.5 nM	Enzyme inhibition	TEM-1	[123]
2010	BLIP-II	Phage display system and *E. coli* expression system	TEM-1: IC${}_{50}$ = 320 pM, TEM-1: $K_{i}$ = 2.5 pM, SME-1: $K_{i}$ = 8.4 pM, SHV-1: $K_{i}$ = 12 pM, Bla1: $K_{i}$ ≤ 25 pM, CTX-M14: $K_{i}$ = 9.1 pM, PC1: $K_{i}$ ≤ 16 pM	Phage ELISA, Enzyme inhibition	TEM-1, SHV-1, Bla 1, SME-1, CTX-M14, PC1	[105]
2010	BLIP	*E. coli* expression system	$K_{D}$ = 1.7 nM	Enzyme inhibition	TEM-1	[124]
2011	BLIP-II	*E. coli* expression system	TEM-1: $K_{D}$ = 0.79 pM, PC1: $K_{D}$ = 7.8 pM, SHV-1: $K_{D}$ = 30 pM, Bla1: $K_{D}$ = 1.1 pM	Enzyme inhibition by SFF	TEM-1, PC1, SHV-1, Bla1	[125]
2011	BLIP	Phage display system and *E. coli* expression system	$K_{i}$ = 0.5 nM, $K_{i}$ = 0.4 nM	Phage ELISA, Enzyme inhibition	TEM-1	[126]
2011	BLIP	*E. coli* expression system	TEM-1: $K_{D}$ = 3.2 nM, SHV-1: $K_{D}$ = 1.8 μM	Enzyme inhibition	TEM-1, SHV-1	[127]
2011	BLIP	*E. coli* expression system	$K_{i}$ = 350 nM, $K_{D}$ = 380 nM, $K_{on}$ = 1×10${}^{6}$ M${}^{-1}$·s${}^{-1}$	Enzyme inhibition by SFF, ITC	PC1	[128]
2012	BLIP	*E. coli* expression system	$K_{D}$ = 3×10${}^{5}$ M${}^{-1}$·s${}^{-1}$	Enzyme inhibition by SFF	TEM-1	[129]
2012	BLIP	*E. coli* expression system	$K_{on}$ = 2.9×10${}^{5}$ M${}^{-1}$·s${}^{-1}$	Enzyme inhibition by SFF	TEM-1	[130]
2013	BLIP-II	*E. coli* expression system	$K_{D}$ = 76 fM	Enzyme inhibition	KPC-2	[108]
2014	BLIP, BLIP-II	*E. coli* expression system	BLIP: $K_{i}$ = 230 pM, BLIP-II: $K_{D}$ = 1.5 pM	Enzyme inhibition by SFF	TEM-1	[107]
2015	BLIP	Peptide synthesis	BLIP peptide (residue H45-Y53): $K_{i}$ = 58 μM	Enzyme inhibition	TEM-1	[131]
2016	BLIP	*E. coli* expression system	TEM-1: $K_{i}$ = 0.5 nM, KPC-2: $K_{i}$ = 1.2 nM, SHV-1: $K_{i}$ = 1.1 μM, CTX-M14: $K_{i}$ = 810 nM, SME-1: $K_{i}$ = 2.4 nM	Enzyme inhibition	TEM-1, KPC-2, SHV-1, CTX-M14, SME-1	[87]
2016	BLIP	*E. coli* expression system	$K_{D}$ = 28 nM	Enzyme inhibition by SFF, SPR	TEM-1	[109]
2017	BLIP	*E. coli* expression system	TEM-1: $K_{i}$ = 0.5 nM, SHV-1: $K_{i}$ = 1.13 μM, KPC-2: $K_{i}$ = 1.5 nM, Bla1: $K_{i}$ = 2.5 nM	Enzyme inhibition	TEM-1, SHV-1, KPC-2, Bla1	[93]
2018	BLIP	*E. coli* expression system	$K_{D}$ = 0.57 nM	Enzyme inhibition by SFF, SPR	TEM-1	[110]

* ELISA: enzyme-linked immunosorbent assay, FQT: fluorescence quenching titration, ITC: isothermal titration calorimetery, SFF: stopped flow fluorometry, SPR: surface plasmon resonance.
